# 

**Published:** 1906-05-12

**Authors:** 


					/
the hospital. jlLJEf May 12th 190G-
TAL
No. 1,024. -Yol. XL. La Newspaper.] SATURDAY,
May 12th 1906
[ aee^nJerms] PRICE THREEPENCE

				

